# Optimal reproduction of a porcine benign biliary stricture model using endobiliary radiofrequency ablation

**DOI:** 10.1038/s41598-022-16340-x

**Published:** 2022-07-14

**Authors:** Chang-Il Kwon, Sung Ill Jang, Seok Jeong, Gwangil Kim, Tae Hoon Lee, Jae Hee Cho, Ji Hae Nahm, Min Je Sung, Kwang Hyun Ko

**Affiliations:** 1 Digestive Disease Center, CHA Bundang Medical Center, CHA University School of Medicine, Seongnam, Republic of Korea; 2 Department of Internal Medicine, Gangnam Severance Hospital, Yonsei University College of Medicine, Seoul, Republic of Korea; 3 Division of Gastroenterology, Department of Internal Medicine, Inha University School of Medicine, Inha University Hospital, 27 Inhang-ro, Jung-gu, Incheon, 22332 Republic of Korea; 4 Department of Pathology, CHA Bundang Medical Center, CHA University School of Medicine, Seongnam, Republic of Korea; 5 Department of Internal Medicine, SoonChunHyang Cheonan Hospital, Soonchunhyang University College of Medicine, Cheonan, Republic of Korea; 6 Department of Pathology, Gangnam Severance Hospital, Yonsei University College of Medicine, Seoul, Republic of Korea

**Keywords:** Gastroenterology, Gastrointestinal models

## Abstract

The use of endobiliary radiofrequency ablation (RFA) to generate a benign biliary stricture (BBS) model has a significant reproducibility problem. The aims of this animal study were to create an optimal BBS model using endobiliary RFA and determine the best way to develop it. The first step was performed on the common bile duct (CBD) of 10 miniature pigs using endoscopic RFA with a target temperature-controlled mode (80 ℃, 7 W for 90 s). The second step was performed on the CBD of five miniature pigs to understand more about the time-dependent changes in BBS development and the causes of adverse events. Using the conditions and techniques identified in the previous steps, the third step was conducted to create an optimal BBS model in 12 miniature pigs. In the first trial, four out of 10 animals died (40%) after the procedure due to cholangitis-induced sepsis. Based on this, biliary obstruction was prevented in further steps by placing a biliary plastic stent after RFA application. Histologic examinations over time showed that a severe abscess developed at the RFA application site on the fifth day, followed by fibrosis on the tenth day, and completion on the twentieth day. In the third trial, 11 animals survived (91.7%), the average BBS fibrotic wall thickness was 1107.9 µm (763.1–1864.6 µm), and the degree of upstream biliary dilation was 14.4 mm (11.05–20.7 mm). In conclusion, endobiliary RFA combined with a biliary plastic stent resulted in a safe and reproducible BBS animal model.

## Introduction

Endoscopic treatment for resolving a malignant biliary stricture (MBS) or benign biliary stricture (BBS) has been continuously developed over the past half-century, and with this development, plastic and metal stents have also been developed and improved, allowing them to be used more effectively and widely. Recently, endoscopic accessories that can provide local therapy directly to the bile duct have been developed, and some of these products are now in use. Before these new or improved products are used in clinical practice, safety should be evaluated as a preclinical evaluation step and whether their effectiveness is superior to existing products should be determined. For this purpose, bile duct models using large animals have been developed.

The animal bile duct model can be divided into two categories. A bile duct dilation model is used to mount the stent in the bile duct to observe the safety or patency of the stents^1–4^. The other is a BBS model used to demonstrate the superiority of new stents or determine the safety and effectiveness of endoscopic accessories for biliary local therapy^5–7^. Both methods have a great burden in terms of cost because large animals must be used. Among them, the BBS model must be implemented very precisely because it has to artificially induce stenosis in the bile duct, and there is a risk associated with inducing complete bile duct obstruction. Also, the ability to obtain reproducible results under the same procedure conditions is limited.

Previous BBS models were implemented in rats through surgical ligation or clamping, but these methods were ineffective for the above-mentioned purposes^8–11^. By directly applying thermal injury to the bile duct with a heat probe or electro-cautery probe, a BBS model using a large animal model was introduced^12,13^. Then, as a more precise and sophisticated method, the large animal BBS model was implemented using endobiliary radiofrequency ablation (RFA)^5^. By delivering heat energy through an RFA probe, necrosis is induced in the tissue around the probe, and a stricture is induced in the damaged tissue through the healing process^14,15^. Several large animal studies confirmed that the BBS model using RFA was implemented safely and effectively, and showed the possibility of its use for various purposes^6,7,16–18^.

The number of complete biliary obstructions and adverse events that occur when BBS is induced too strongly, and how to solve these issues is unknown. In addition, when producing a BBS model under the previously reported conditions, the reproducibility needed to obtain an appropriate BBS status to the desired degree has not yet been demonstrated. Furthermore, by observing the step-by-step changes that cause BBS through time-sequential analysis, it is important to determine when to conduct the main experiment for a given purpose. Because upstream biliary dilation occurs in stages as BBS progresses, it is also important to determine when the main experiment should be conducted to minimize adverse events^7^. If the aforementioned issues are not objectively addressed, there will inevitably be significant limitations in the use of BBS animal models for preclinical research, which is very expensive.

The purpose of this porcine animal study was to create an optimal BBS model using endobiliary RFA and determine the best way to develop it for high reproducibility.

## Materials and methods

### Animals and pre-procedure preparation

Fourteen-month-old miniature pigs (*Sus scrofa*), each weighing approximately 25–33 kg (Cronex Co., Ltd., Hwaseong, Republic of Korea), were used for the in vivo experimental study. The animals were kept in specific pathogen-free animal facilities with complete substrate feeding, according to standard guidelines for laboratory animals. All animals were quarantined and acclimated in a vivarium for seven days before the experiments. The animals were kept in a specific pathogen-free animal research facility (KNOTUS Co., Ltd., Songdo, Republic of Korea) with complete substrate feeding, according to standard guidelines for laboratory animals. The animals were fasted overnight before the endoscopic procedure, but water was provided ad libitum. Pre-anesthesia sedation was conducted, which consisted of an intramuscular injection of atropine sulfate (0.04 mg/kg), xylazine (2 mg/kg), and tiletamine-zolazepam (5 mg/kg). Subsequently, the pigs were intubated, and general anesthesia was achieved using 0.5% to 2% isoflurane through an endotracheal tube with 70% nitrous oxide and 30% oxygen provided by a ventilator. The pigs were then placed on their left lateral side on a fluoroscopy table.

### RFA equipment and instruments

**Figure 1:**
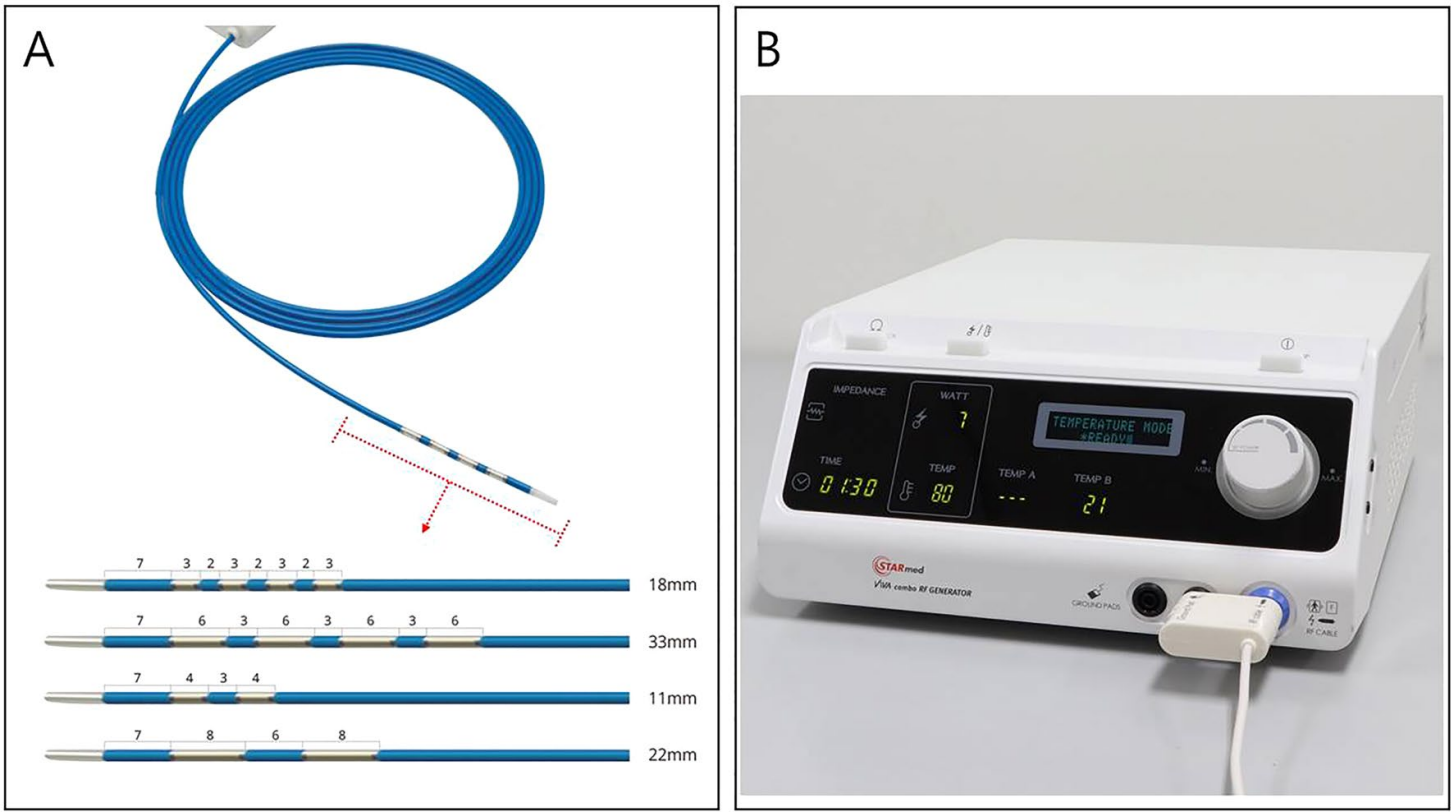
The endobiliary radiofrequency catheter (ELRA™ RF catheter, STARmed, Goyang, Korea) and power generator (VIVA Combo™; STARmed) used for endobiliary RFA (Courtesy of STARmed). (**A**) An endobiliary RFA catheter and the distal ends of different types of RFA catheters. Among them, the 7 ~ 10-W/22-mm type was used in this study. (**B**) The radiofrequency power generator and our settings.

An endobiliary RFA catheter (7F, 175-cm working length; ELRA™ RF catheter, STARmed, Goyang, Korea) was used for the study (Fig. 1A). Among different electrode types, the 7 ~ 10-W/22-mm type was used. Radiofrequency energy was delivered by a RFA generator (VIVA Combo™; STARmed) (Fig. 1B) in a target temperature-controlled mode (80 ℃, 7 W for 90 s), which could automatically terminate ablation if a certain preset temperature was exceeded. The RFA setting conditions were set to the safe values identified in previous reports^6,7,16^.

### Endoscopic procedure

**Figure 2:**
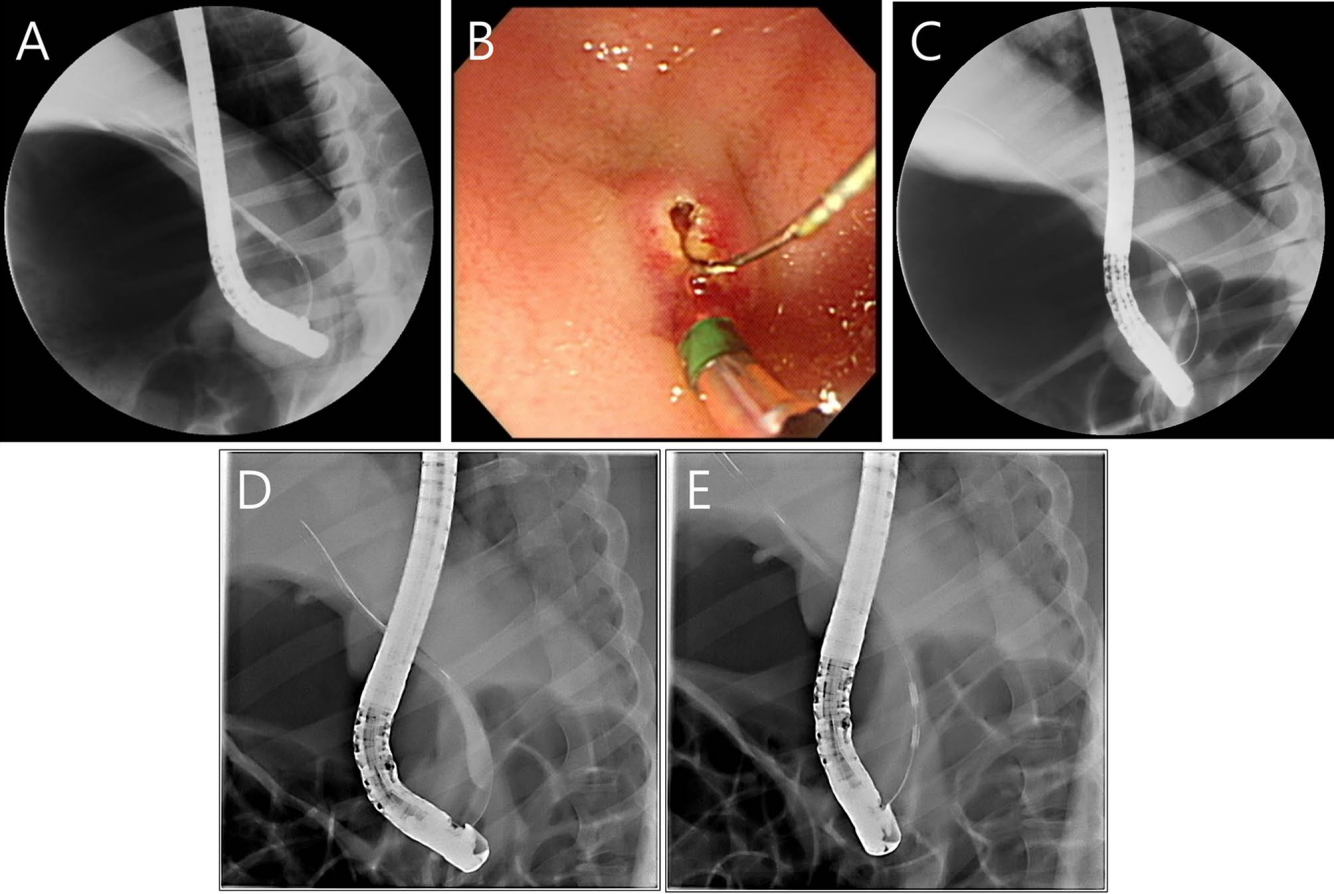
Endoscopic retrograde cholangiography examination of a miniature pig during endobiliary RFA. (**A**) Cholangiogram shows non-dilated CBD before endobiliary RFA. (**B**) Endoscopic biliary sphincterotomy. (**C**) Fluoroscopic examination shows the RFA catheter positioned in the distal CBD while performing RFA. (**D**) and (**E**) Fluoroscopic views show the suction technique as in vacuum evacuation to maximize the contact between the CBD mucosa and the RFA catheter, even if the CBD was dilated.

Expert biliary endoscopists (C-I. K, SI. J., and S. J.) performed endoscopic retrograde cholangiography (ERC) on the animals using a standard side-viewing duodenoscope (TJF-240; Olympus Co. Tokyo, Japan). After duodenal intubation with the scope, an endoscopic retrograde cholangiopancreatography (ERCP) sphincterotome (CleverCut3V™; Olympus Co) was inserted into the bile duct using the wire-guided cannulation technique with a 0.035-inch hydrophilic-tipped guidewire (Boston Scientific Corporation, Natick, MA, USA) to obtain a cholangiogram (Fig. 2A). Then, endoscopic biliary sphincterotomy was done (Fig. 2B) for the easy insertion of the RFA catheter and a suction technique was used to maximize contact between the mucosa of the CBD and the RFA catheter. The endobiliary RFA catheter was advanced into the distal common bile duct (CBD) over the guidewire under fluoroscopic guidance (Fig. 2C). RFA was performed with the previously mentioned settings using the suction technique (Fig. 2D,E). If the RFA catheter did not contact the mucosa of the CBD, the temperature displayed on the RFA generator did not reach the target point and the power was automatically turned off. Additional post-RFA cholangiography was performed to evaluate any immediate adverse events such as perforation or bleeding.

### Three-step *in-vivo* animal study to evaluate study outcomes

**Figure 3:**
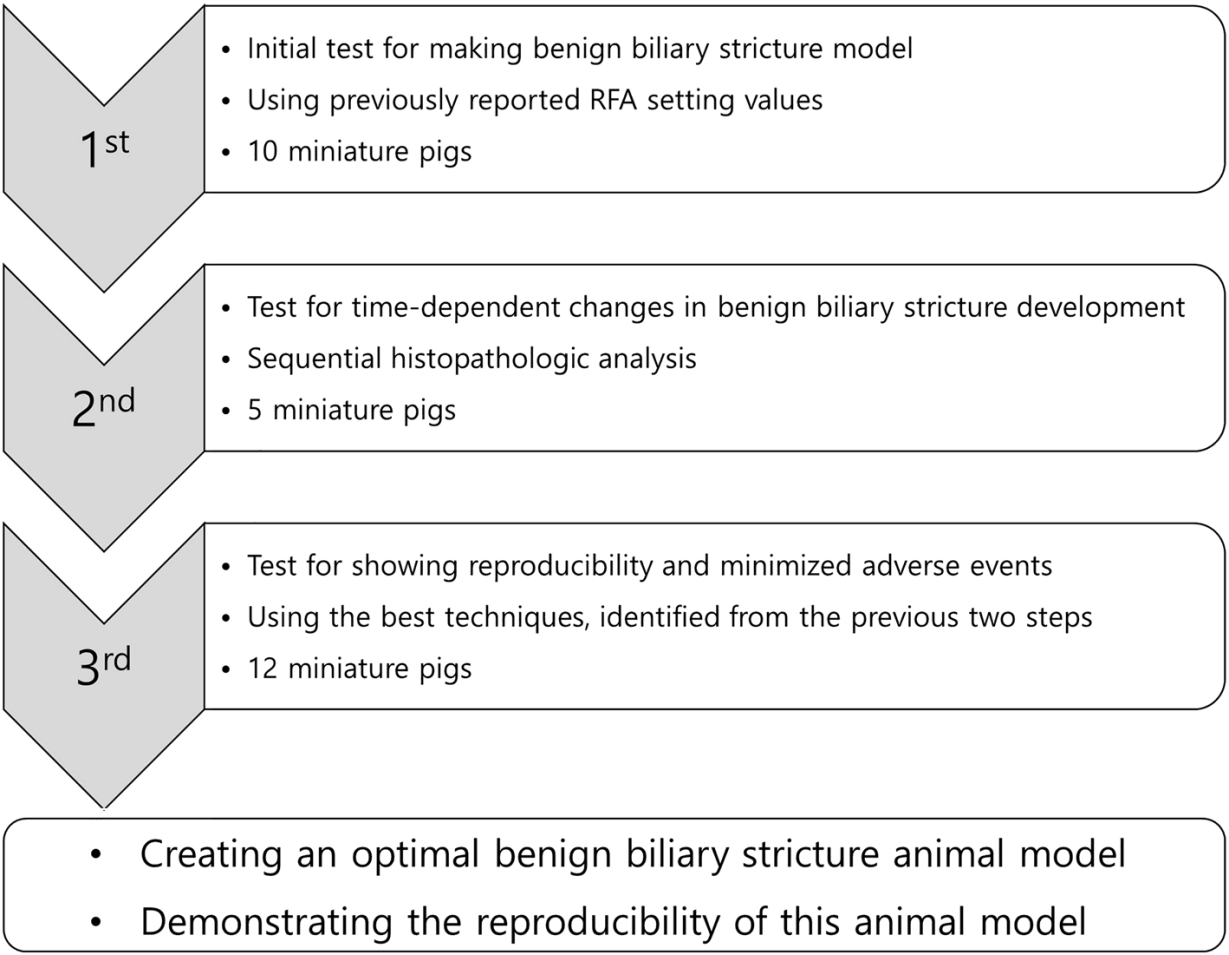
Schematic diagram of the experimental steps for the study outcomes.

We designed and conducted this large animal study in three steps (Fig. 3). (1) The first step was performed on the distal CBD of 10 pigs, which was an initial test for making a benign biliary stricture model using endoscopic RFA with the previously reported RFA setting values. This step was to objectively evaluate whether an adequate BBS animal model was generated by applying the previously reported RFA setting values, as well as how many adverse events occurred. (2) The second step was performed on the distal CBD of five animals using endoscopic RFA with the same settings. This step was a sequential histopathologic analysis to understand more about the time-dependent changes in BBS development and the causes of the adverse events. This step was to track the changes in CBD tissue over time after RFA application and determine exactly when and how stricture-induced histological changes occurred. (3) The third step was carried out to create an optimal BBS model for 12 animals using endoscopic RFA with the same settings and identify the best techniques from the previous two steps. In this step, referring to the information found in the previous two steps, the appropriate conditions and techniques for generating the BBS model were determined, and additional techniques to minimize adverse events were applied. This was a step to finally confirm whether the BBS animal model could demonstrate acceptable reproducibility.

### Post-procedure follow-up and histological examination

During the follow-up period after the procedure, the pigs were fed their usual diet. Clinical signs and parameters including weight loss, daily food intake, and demeanor scores were monitored daily. Laboratory tests including liver function tests were assessed at baseline and at regular intervals during the follow-up period to evaluate cholangitis and biliary obstruction.

When the scheduled time was reached during the follow-up period, ERCP was repeated to identify the BBS and adverse events. Adequate BBS was defined as a luminal diameter decrease of more than 50% from baseline and the presence of upstream bile duct dilation. Then, the animal was euthanized immediately by potassium chloride overdose, and the CBD was obtained to evaluate pathologic findings. With the exception of serious adverse events, the pigs were euthanized at the scheduled time after the procedure. A serious adverse event was defined as an event that led to death or serious deterioration in health (life-threatening illness or injury, permanent impairment of bodily structure or function).

A laparotomy was performed under general anesthesia, and the CBD, and adjacent duodenum were extracted. The stricture formation and the effect of RFA on the distal CBD, and upstream CBD dilation were assessed macroscopically during necropsy.

Histopathologic examination of formalin-fixed, paraffin-embedded CBD specimens was performed with hematoxylin & eosin and Masson’s trichrome stain and examined under light microscopy by an experienced pathologist (GK). Inflammatory reactions in the mucosa, and the degree of submucosal fibrosis were analyzed. The slides were scanned to obtain digital images using a PANNORAMIC 250 scanner (3DHistech, Budapest, Hungary) and fibrotic wall thickness was measured by CaseViewer software (3DHistech).

### Statistical analysis

The median and range were used to summarize the data for the continuous variables. The differences between the groups were analyzed using the Mann–Whitney U test. *P* < 0.05 was considered statistically significant. Statistical analyses were performed with IBM® SPSS® Statistics (Version 21.0.0; SPSS Inc., Chicago, IL, USA).

### Ethics statement

All stages of this animal experiment were approved by the Institutional Animal Care Committee of KNOTUS Co., Ltd., (KNOTUS IACUC: 19-KE-468, 20-KE-588, and 20-KE-654). All experimental procedures were conducted in accordance with the guidelines of the ethics committee and the stipulations in the manuscript. This study was carried out in compliance with the Animal Research: Reporting of In Vivo Experiments (ARRIVE) guidelines.

## Results

### Results of the first *in-vivo* study step

**Figure 4:**
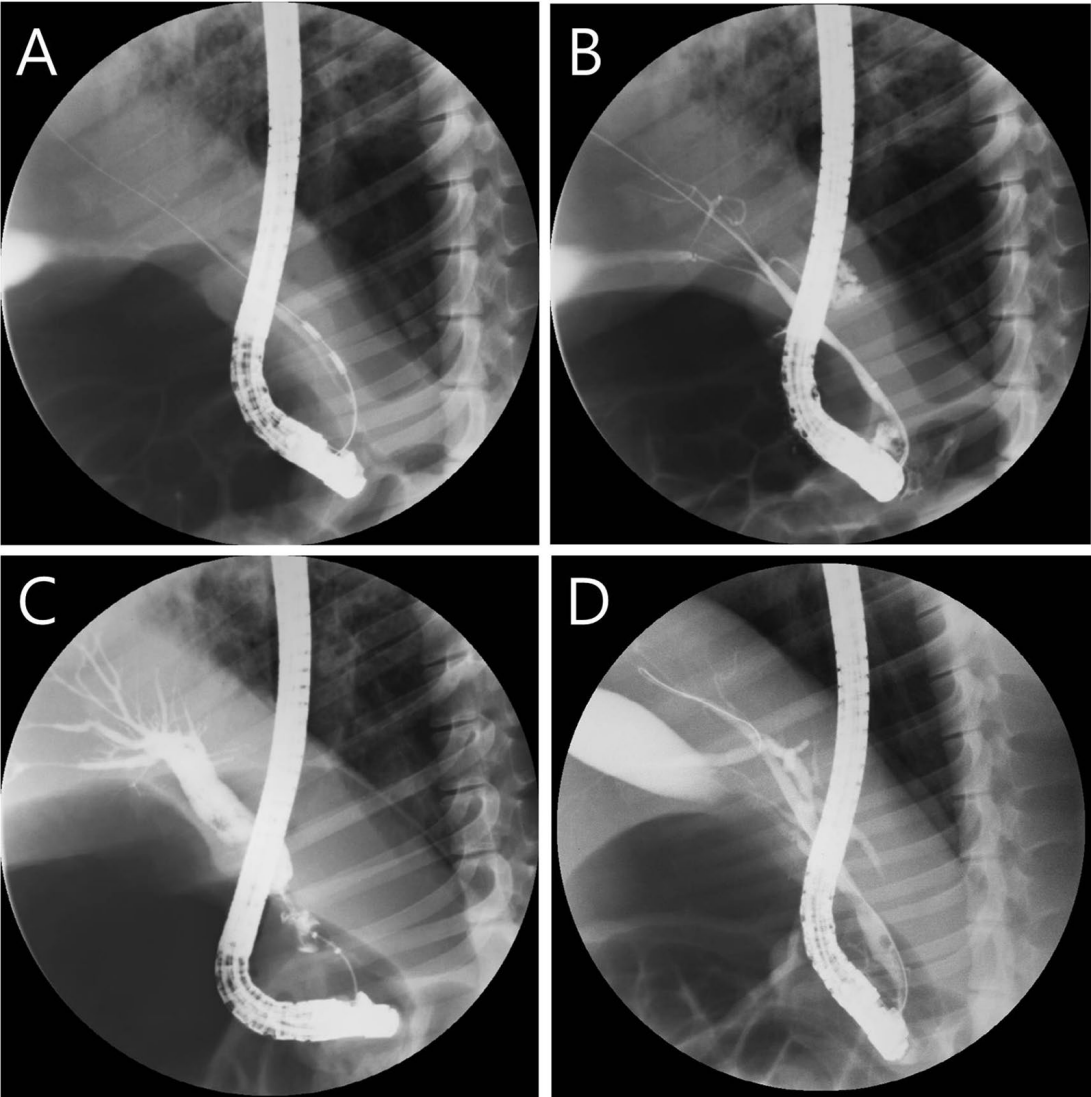
Endoscopic retrograde cholangiogram of miniature pigs. (**A**) Endobiliary RFA was performed in the distal common bile duct. (**B**) Immediate post-RFA cholangiogram showed no contrast leakage. (**C**) Follow-up cholangiogram at four weeks showed adequate post-RFA biliary stricture and definite upstream duct dilation. (**D**) Another representative follow-up cholangiogram at four weeks showed inadequate post-RFA biliary stricture and mild upstream duct dilation.

After endoscopic RFA application using previously reported settings, four out of 10 animals died unexpectedly (40%) between two and four weeks after the procedure. We proceeded with immediate autopsies to determine the cause of death and found that all four animals died of sepsis due to cholangitis. In the six surviving animals, ERC was obtained four weeks after the procedure to confirm the degree of BBS (Fig. 4). In one animal (No. 5), BBS was not formed, probably due to inadequate RFA application (Fig. 4D). On the ERC examination of five animals, the median length of the stricture area was 10.1 mm (7.1–12.9 mm), and the median diameter of the upstream dilation was 20.6 mm (8.1–33.8 mm) (Table 1).

**Table 1:** Summary of the first *in-vivo* animal study step.

ID	Follow-up for 4 weeks	Endoscopic retrograde cholangiography			
		Technical success	Contrast leakage	Stricture length (mm)	Upstream dilation (mm)
1	(−), died at 10 days	n/a	n/a	n/a	n/a
2	(−), died at 35 days	n/a	n/a	n/a	n/a
3	( +)	( +)	(−)	9.5	8.1
4	( +)	( +)	(−)	10.1	33.8
5	( +),	(-)	(−)	n/a	n/a
6	( +)	( +)	(−)	7.1	8.4
7	(−), died at 14 days	n/a	n/a	n/a	n/a
8	(−), died at 3 days	n/a	n/a	n/a	n/a
9	( +)	( +)	(−)	12.9	26.4
10	( +)	( +)	(−)	11.9	20.6
Median value				10.1 (7.1–12.9)	20.6 (8.1–33.8)

n/a, not applicable.

* Circumference of the transverse axis at the widest upstream biliary dilation area.

### Results of the second *in-vivo* study step

We planned to euthanize one animal every five days after performing endobiliary RFA on five animals and perform sequential histological examinations. Before the histological examination, gross examination to evaluate CBD rupture, perforation, bleeding and BBS degree was performed. Based on the results of the first experimental trial, we had to prevent BBS-induced biliary obstruction and secondary cholangitis. Therefore, a plastic stent (single pigtail-type, 5 Fr, 7 cm, with flap: Zimmon® Pancreatic Stent, Cook Medical, Bloomington, IN, USA) was placed for biliary drainage after RFA application.

However, one animal died unexpectedly on the fourth day of follow-up, and the immediate autopsy to determine the cause of death showed no overt bleeding or perforation in the CBD. Because blood tests could not be performed due to the sudden death, the exact cause of death could not be determined. However, the animal ate very little food, the body condition deteriorated, the previously inserted plastic stent was not examined in the CBD, and the body weight dropped from 28.2 to 25.6 kg in four days. Therefore, death was presumed to be due to a septic condition. Therefore, the remaining animals were administered cefazolin (Cefozol; Koruspharm, Chuncheon, Korea) 25 mg/kg intravenously (IV) every eight hours during the follow-up. No specific clinical symptoms, abnormal laboratory findings, or adverse events were observed during the follow-up period in the other four animals.

**Figure 5:**
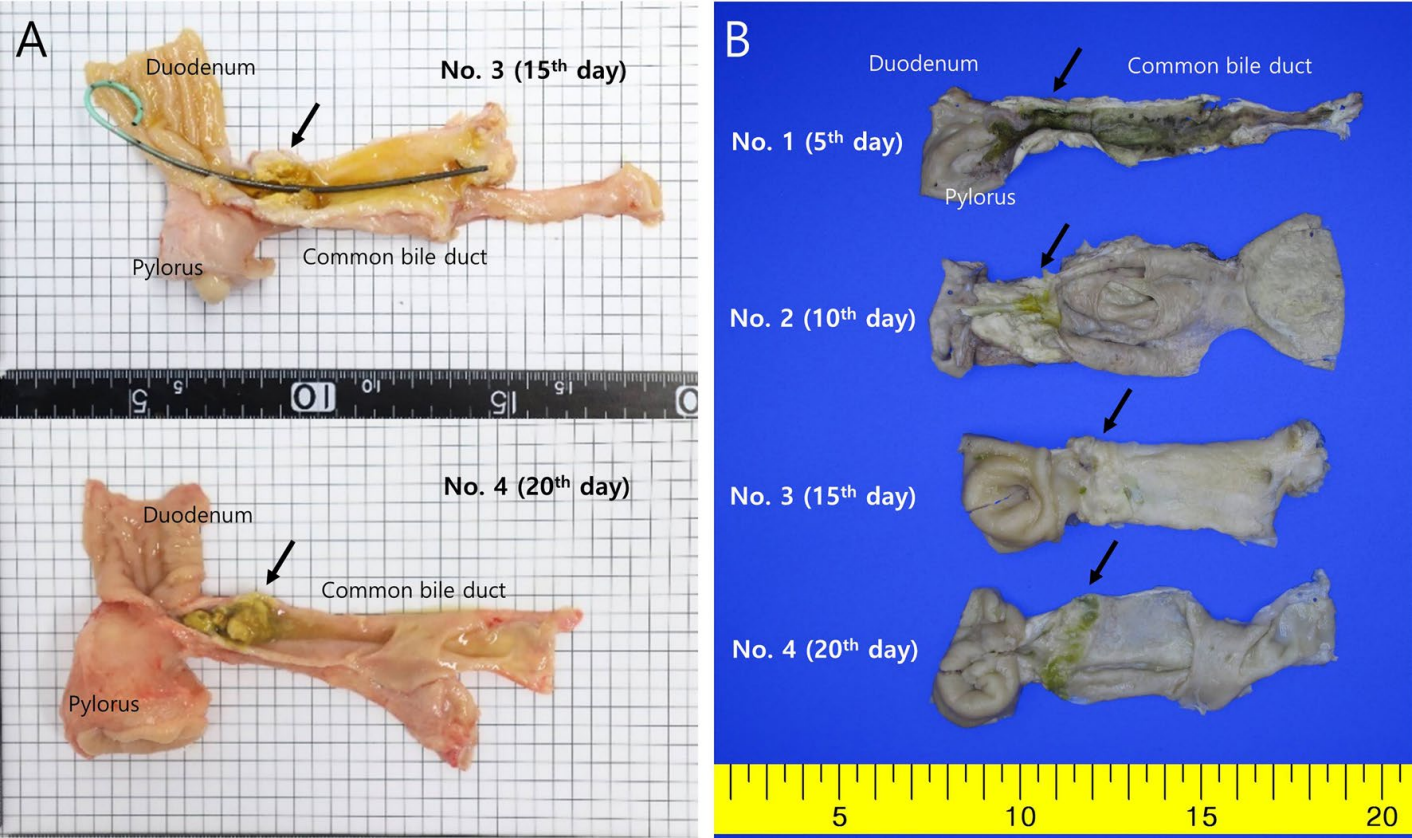
Histopathologic gross images of resected and longitudinally opened CBD. (**A**) Gross images of the common bile ducts, exposed immediately after excision. (**B**) Gross images of the common bile ducts after formalin fixation. Each APC application area (arrow) became thicker and more prominent over time in the third and fourth animals.

**Figure 6:**
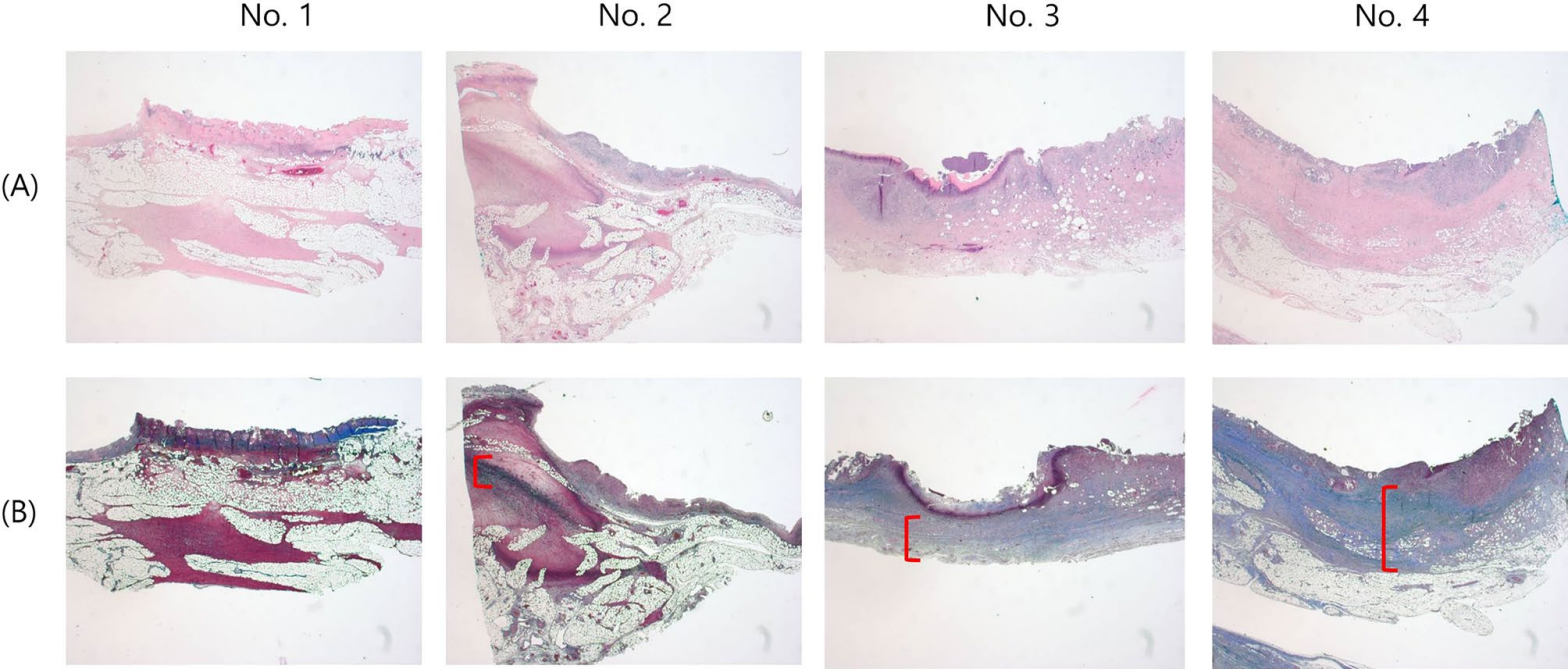
Histopathologic microscopic images of the APC application area of the CBD. Extensive abscesses replace the thickened wall at the APC application area of the first and second animals. Fibrosis is observed to begin in the second animal. In the third and fourth animals, abscess formation was greatly decreased, and the thickened wall is nearly replaced by fibrosis (parentheses indicate fibrosis thickness). (**A**). H&E; original magnification 40 × . (**B**) MT; original magnification 40 × .

The remaining four animals were euthanized 5, 10, 15, and 20 days after the procedure. On macroscopic examination, the inserted plastic stent was visible in only two out of four animals (Fig. 5A). Perforation or other adverse events were not observed in any of the four animals. The stricture initially was mild in the RFA application area in the distal CBD of the first animal, and the stricture and upstream CBD dilation were clearly observed in the second animal (Fig. 5B). Those areas became thicker and more prominent over time in the third and fourth animals. On microscopic examination (Fig. 6), the CBD of the first animal demonstrated extensive abscess formation at the APC application area, but no fibrosis was found. In the CBD of the second animal, the majority of the wall was replaced with an abscess at the APC application area, but fibrosis had begun, as evidenced by Masson's trichrome stain. In the third and fourth animals, the abscess formation greatly improved, and the thickened wall was nearly and completely replaced by fibrosis.

### Results of the third *in-vivo* study step

**Figure 7:**
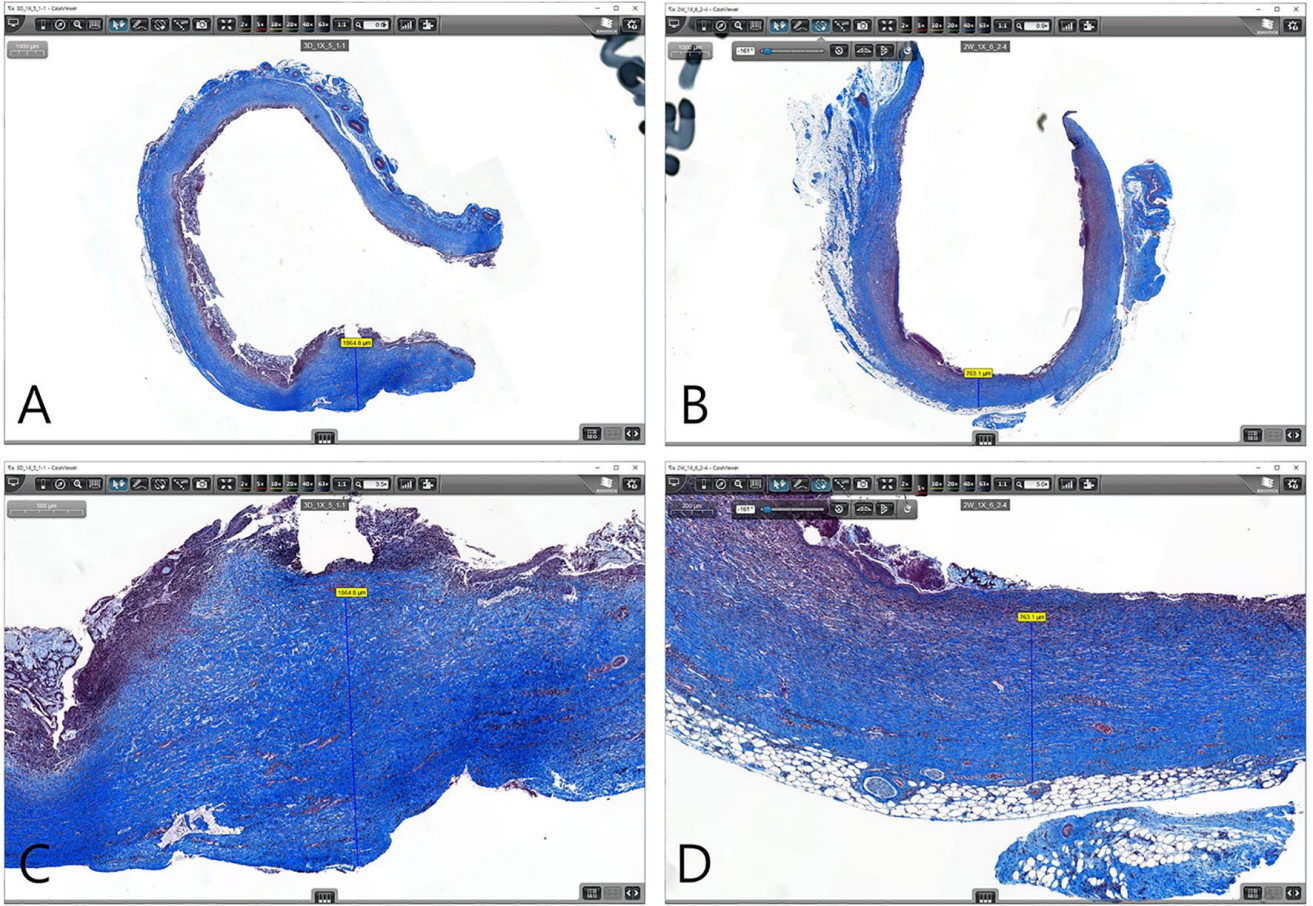
Measurement of periductal fibrosis (fibrous wall thickness). The thickness of bile duct fibrosis was measured using the distance measurement tool in CaseViewer software (3DHistech). The thickest (**A**) and the thinnest areas (**B**) were measured in a region of dense collagen deposition under the muscle layer by Masson’s trichrome staining. (**C**) Magnified image of (**A**). (**D**) Magnifified image of (**B**).

Based on the previous steps and the objective to prevent stent migration, biliary plastic stents were replaced with double-pigtail-type (5Fr, 9 cm: Zimmon® Biliary Stent, Cook Medical) and inserted after RFA in 12 animals. The proximal ends of the stents did not return to their original shape, indicating that they had an anchoring effect within the CBD. During the follow-up period, one out of 12 animals died unexpectedly (8.3%) 13 days after the RFA procedure. The autopsy revealed that there was no perforation or necrosis. In the 11 surviving animals, blood analyses showed mild cholangitis during the two weeks after RFA but no cholangitis four weeks after RFA (Table 2). ERC was obtained four weeks after the procedure to confirm the degree of BBS. Stent migration occurred in only one out of 11 animals (9.1%). The median length of the stricture area was 27.0 mm (17.4–30.9 mm), and the median diameter of the upstream dilation was 14.4 mm (11.1–20.7 mm) (Table 3). On histopathologic examination (Fig. 7), the average fibrotic wall thickness of the BBS was 1107.9 µm (763.1–1864.6 µm).

**Table 2:** Laboratory findings during the third *in-vivo* animal study step.

Variables Period (mean ± SD)	Pre-RFA	1 week after RFA	2 week after RFA	4 week after RFA
WBC (× 10^3^cells/µℓ)	16.47 ± 4.03	22.81 ± 5.09†	29.47 ± 9.73†	21.83 ± 10.21
RBC (× 10^6^cells/ µℓ)	6.89 ± 0.42	7.39 ± 1.33	6.64 ± 1.65	4.79 ± 0.60†
Hb (g/dL)	11.53 ± 1.27	12.60 ± 2.64	11.23 ± 3.32	7.57 ± 2.24†
PLT (× 10^3^ cells/ µℓ)	409.00 ± 108.09	343.36 ± 113.68	417.00 ± 145.39	529.36 ± 224.67
ALP (U/L)	309.36 ± 77.25	342.93 ± 192.13	361.95 ± 183.67	405.95 ± 245.56
ALT (U/L)	55.40 ± 19.59	34.75 ± 12.06	25.39 ± 9.17†	23.85 ± 8.67†
AST (U/L)	54.19 ± 33.56	109.89 ± 97.62	70.65 ± 29.75	69.20 ± 32.11
T. Bil (mg/dl)	0.39 ± 0.26	1.56 ± 1.77†	2.95 ± 2.41†	1.64 ± 1.54†

RFA, radiofrequency ablation; SD, standard deviation; WBC, white blood cells; RBC, red blood cells; Hb, hemoglobin; PLT, platelet; ALP, alkaline phosphate; ALT, alanine transaminase; AST, aspartate transaminase; T.Bil, total bilirubin.

^†^, *P* < 0.005 compared to Pre-RFA.

**Table 3:** Summary of the third step *in-vivo* animal study step.

ID	Follow-up for 4 weeks	Endoscopic retrograde cholangiography					Histopathologic evaluation
		Technical success	Contrast leakage	Stent migration	Stricture length (mm)	Upstream dilation (mm)	Fibrotic wall thickness (µm)
1	( +)	( +)	(−)	( +)	19.4	11.8	895.7
2	( +)	( +)	(−)	(−)	23.6	18.7	970
3	( +)	( +)	(−)	(−)	30.2	14.4	1107.9
4	(−), died at 13 days	n/a	n/a	(−)	n/a	n/a	n/a
5	( +)	( +)	(−)	(−)	28.8	16.2	1864.6
6	( +)	( +)	(−)	(−)	28.7	11.8	1418.5
7	( +)	( +)	(−)	(−)	17.4	17.1	763.1
8	( +)	( +)	(−)	(−)	22.8	11.1	1257.9
9	( +)	( +)	(−)	(−)	19.3	11.6	1200.0
10	( +)	( +)	(−)	(−)	30.9	15.4	1378.6
11	( +)	( +)	(−)	(−)	27.0	13.5	883.1
12	( +)	( +)	(−)	(−)	29.9	20.7	931.6
Median value					27.0 (17.4–30.9)	14.4 (11.1–20.7)	1107.9 (763.1–1864.6)

n/a, not applicable.

* Circumference of the transverse axis at the widest upstream biliary dilation area.

## Discussion

We conducted a three-step animal study and found that when an animal BBS model was created using endobiliary RFA, a previously reported method, there were serious adverse events and a high probability of failure. The process of BBS formation was first examined histopathologically, and several methods to prevent adverse events were added to create an optimal BBS model using endobiliary RFA. Eventually, through this study, we demonstrated its reproducibility.

Ideal animal models used for research have challenging problems of high price and low reproducibility, so small animals such as mice and rats are mainly used. However, there is a significant problem in that endoscopic procedures and endoscopic devices must be directly verified, necessitating the use of large animal models in those studies. In terms of cost, this inevitably becomes a burden for researchers and device manufacturers. As mentioned in the Introduction, to determine the effect of a biliary stent or device, a model for improving BBS must be primarily used. The merit is greater in terms of cost if the procedure is performed directly on the animal, but the cost becomes a bigger problem because the test stent or device must be evaluated after the model is artificially created. Since previous animal BBS models using endobiliary RFA have already been demonstrated to some extent, our main purpose was to understand the process of making a BBS and add several new processes to improve the reproducibility.

According to the results in the first step, unexpected sudden death occurred in 40% of the pigs before the BBS model was created. This is a critical and important issue indicating that this animal experimental model could not be used. The cause of death was a problem, with CBD drainage prior to the onset of the BBS, which resulted in cholangitis-induced sepsis. The ERC test performed on the surviving animals showed that the degree of BBS was so severe that the guidewire was not inserted in one case. As suggested in a previous study, since a BBS caused by RFA is induced slowly, it seemed that sudden CBD obstruction would not be a problem. However, in practice, critical problems occurred before the BBS was completely formed^7^.

Based on the results of the first step, we placed a plastic stent to prevent BBS induced biliary obstruction and secondary cholangitis in the second-step trial. As shown in all clinical studies on intraductal RFA in malignant biliary obstruction, a stricture is an unconditional adverse event after RFA. Therefore, biliary stenting with a plastic or metal stent was performed in all patients to prevent this occurrence^19–22^. Based on this, we tried inserting a plastic stent for the same purpose. However, one animal with early stent migration died suddenly on the fourth day, and stent migration was also observed in two animals during follow-up. Perhaps, unlike a malignant biliary stricture, since a plastic stent was inserted before a benign stricture was formed, stent migration may have easily occurred. On microscopic examination (Fig. 6), it was found that an abscess had formed on the entire wall of the APC application area as the result of severe inflammation at the beginning of the procedure. BBS-induced cholangitis may cause sepsis, but sepsis could also be caused by abscess formation in the CBD wall. As a result, it was critical to administer broad-spectrum antibiotics during the follow-up period, and the animals’ unexpected sudden death could be avoided. In addition, it was found that fibrosis at the APC application site began on the tenth day and was completed by about the 20th day. This finding suggests that this BBS animal model can be used three weeks after RFA application to the CBD.

Based on the results of the previous two steps, both pigtail-type stents were replaced to prevent stent migration. The biggest concern with adding this step was that as the stent is inserted, stricture and upstream dilation may not be created as expected. Because stent migration occurred at four weeks in one out of 11 pigs (9.1%), which was lower than we expected, it is thought that unexpected sudden death could be prevented by good stent maintenance in the early follow-up period. According to the laboratory tests (Table 2), obstructive cholangitis did occur even if the stent had not migrated from the CBD. As we presumed, stent function was well-maintained at an early stage to prevent sudden death, but the stent function became problematic after two weeks due to the thin diameter of the stent. As a result, while this induced less upstream CBD dilation, there was no problem in maintaining survival for four weeks due to the administration of antibiotics. Upstream CBD dilation is a phenomenon that accompanies the formation of a BBS and is not a necessary result. However, it would be easier to insert the test device or stent when dilation is formed wider than the original diameter.

Based on the new findings of this three-step study, we would like to present guidelines for creating an optimal benign biliary stricture animal model (Table 4). Because RFA settings differ depending upon the RFA generator and probe used, the guidelines for RFA settings are limited. However, it is thought that if the electrode length is too long, there is a high risk of adverse events occurring before the BBS is formed. In contrast, if the electrode length is too short, it may be difficult to use it to assess the treatment effect of stents or devices used for BBS. For the above reasons, we recommend a length of about 20 mm. To ensure maximum contact between the probe and the CBD mucosa during RFA application, a biliary sphincterotomy in advance, and endoscopic suction are recommended. If the initial CBD diameter is not wide, the RFA probe could easily contact the CBD wall. Therefore, biliary sphincterotomy might not be necessary, and rather, promote the migration of plastic stents. Additional tips for preventing adverse events include administering intravenous broad-spectrum antibiotics during the follow-up period, inserting biliary plastic stents after RFA, and considering a double-pigtail-type stent to prevent stent migration.

**Table 4:** Our guidelines for creating an optimal benign biliary stricture animal model.

1. RFA settings
- Electrode length: 18–22 mm - Watt: 7–10 W - Duration: 90–120 s - Mode: Target temperature-controlled mode (80–100℃) * Our settings: 22 mm / 7 W / 90 S / 80℃
2. Creating the method and techniques
- Obtaining initial cholangiogram - Endoscopic biliary sphincterotomy : Not necessary if the initial CBD diameter is not wide - Inserting RFA catheter and positioning at the mid to distal common bile duct - Keeping endoscopic suction during RFA - Obtaining post-RFA cholangiogram
3. Main procedure timing after RFA
- At least 3 weeks after RFA
4. Additional tips for preventing adverse events
- Administration of intravenous broad-spectrum antibiotics during the follow-up period - Inserting biliary plastic stents after RFA Consider double-pigtail-type stent for preventing stent migration 5F or 7F in diameter and > 7 cm in length

RFA, radiofrequency ablation; CBD, common bile duct.

The limitations of this experimental study were as follows. 1) This study used an in vivo animal model, and the sample size was small. 2) RFA settings differed depending upon the RFA generator and probe used. 3) In the unexpected death of animals, it was necessary to guess the cause of death as an autopsy finding due to that accurate laboratory analyses could not be performed during the autopsy. Nevertheless, although additional large-scaled studies are required to observe the reproducibility of this BBS animal model, our current findings provide basic and standardizing techniques for creating an optimal BBS animal model. Thus, the present study results yielded important insight into the potential benefits of the BBS animal model, which be helpful in the development of a new device, technology, or treatment strategies. Since making a large animal model is difficult and requires very sophisticated techniques, researchers may encounter numerous difficult processes, and unexpected adverse events may occur. It is most important for researchers wanting to make this model learn the methods for minimizing adverse events from well-trained researchers before beginning their work. Nevertheless, we think that the learning curve of researchers can be greatly shortened by referring to the optimal methods we introduced as a result of this study. Also, if the optimal method of making the BBS animal model is well established, it will be helpful in various ways in the future. For example, according to a recently reported animal study, pancreatic duct ligation causes pancreas atrophy and eventually, reduced premalignant pancreatic lesions^23^. If the method of inducing pancreatic atrophy by applying endoluminal RFA to the main pancreatic duct is well established^24^, it will have great potential for use clinically as a pancreatic cancer treatment method.

In conclusion, endobiliary RFA combined with a biliary plastic stent resulted in a safe and reproducible BBS animal model. Also, we first examined the process of BBS formation through time-sequential histopathological examinations. Our detailed experimental results will provide basic information and a basis for future BBS animal model production.

## Supplementary Information

Supplementary Information.

## Data Availability

All data generated or analysed during this study are included in this published article and its supplementary information file.
