# Supplementary material for: Optimal reproduction of a porcine benign biliary stricture model using endobiliary radiofrequency ablation
Source: Sci Rep. 2022 Jul 14;12:12046. doi: 10.1038/s41598-022-16340-x (PMC9283468; doi:10.1038/s41598-022-16340-x)
Supplement: Supplementary file 1 — Supplementary Information. [file 41598_2022_16340_MOESM1_ESM.docx]

Supplementary Table 1. Raw data of Table 2.
